# Effect of stress-induced hyperglycaemia on clinical outcome in paitients with acute ST-segment elevation myocardial infarction undergoing percutaneous coronary intervention

**DOI:** 10.3389/fcvm.2026.1763222

**Published:** 2026-05-14

**Authors:** Kaidong Zeng, Zhixiong Liao, Yanming Du, Jianping Ding

**Affiliations:** Department of Cardiology, Zhangjiajie People’s Hospital, Zhangjiajie, China

**Keywords:** major adverse cardiovascular events (MACE), percutaneous coronary intervention (PCI), prognosis, stress hyperglycemia, ST-segment elevation myocardial infarction (STEMI)

## Abstract

**Background:**

Stress hyperglycemia (SHG) is a transient glycemic disturbance induced by acute stress, and its independent prognostic value in patients with acute ST-segment elevation myocardial infarction (STEMI) undergoing percutaneous coronary intervention (PCI) remains to be fully clarified. This study aimed to investigate the impact of SHG on short- and long-term outcomes in this population and evaluate its role in risk stratification.

**Methods:**

A retrospective analysis was conducted on 818 first-time STEMI patients who underwent emergency PCI between July 2020 and February 2024. Patients were classified into three groups based on diabetes history, fasting blood glucose (FBG) levels, and glycated hemoglobin (HbA1c) levels: 1) SHG group (*n* = 200): Non-diabetic patients with FBG ≥7.0 mmol/L and HbA1c < 6.5%; 2) Non-SHG group (*n* = 319): Non-diabetic patients with FBG <7.0 mmol/L and HbA1c < 6.5%; 3) Diabetes group (*n* = 299): Patients with HbA1c ≥ 6.5% or previously diagnosed diabetes. Major adverse cardiovascular events (MACE) were compared during hospitalization and at one-year follow-up. A multivariate logistic regression model was used to assess the independent association of SHG with in-hospital prognosis, and a Cox proportional hazards model was applied for one-year outcomes.

**Results:**

The SHG group had significantly higher in-hospital all-cause mortality (IHACM; 13.4% vs. 2.19% in the non-SHG group, *P* < 0.001), and Multivariate logistic regression analysis confirmed SHG as the strongest independent risk factor for IHACM (OR = 4.68, 95% CI: 1.98–11.04). At one year, the SHG group showed higher mortality (14.9% vs. 3.76%) and heart failure incidence (26.0% vs. 12.23%, *P* < 0.05) compared with the non-SHG group. However, long-term mortality was primarily associated with Killip classification (HR = 4.67, 95% CI: 2.68–8.15) and coronary artery disease severity (Gensini score, HR = 1.05, 95% CI: 1.03–1.07).

**Conclusions:**

SHG is a strong independent risk factor for IHACM in STEMI patients, with potential mediation by acute metabolic disorders and endothelial injury. Notably, acute hemodynamic instability cannot be excluded as a contributing factor to the observed association, and further studies are needed to clarify the precise mediating pathways. Long-term prognosis is mainly influenced by baseline cardiac function and coronary artery disease severity. Incorporating SHG (defined as FBG ≥7.0 mmol/L in non-diabetic patients with HbA1c < 6.5%) into early risk stratification and developing individualized management strategies for acute-phase glucose fluctuations are recommended.

## 1 Introduction

Stress hyperglycemia (SHG) refers to glucose metabolism disorders characterized by elevated blood glucose levels due to abnormal energy and material metabolism under stress conditions such as severe infection, trauma, massive hemorrhage, or acute poisoning. In the context of acute cardiovascular disease, stress hyperglycemia represents a transient, stress-related elevation of blood glucose and should be clearly distinguished from chronic dysglycemia fulfilling the diagnostic criteria for diabetes mellitus. Acute myocardial infarction (AMI) is a stress state that can trigger SHG. Epidemiological data indicate that the incidence of SHG in patients with acute coronary syndrome at admission ranges from 25% to 50%. Moreover, several large-scale prospective cohort studies have consistently demonstrated that stress hyperglycemia at admission is independently associated with increased risks of in-hospital complications, long-term mortality and heart failure in patients with acute myocardial infarction, highlighting its importance as a prognostic biomarker (1–4). A recent study further confirms that acute glucometabolic markers effectively predict periprocedural myocardial infarction after PCI and long-term adverse cardiovascular events (5). However, the definition of SHG in diabetic patients and its relationship with diabetes remain controversial. Multiple factors influence AMI prognosis, but the specific role of SHG among these factors remains unclear. In recent years, the stress hyperglycemia ratio (SHR), which combines admission blood glucose with the estimated chronic glycemic level derived from HbA1c, has been proposed as a more refined indicator of acute glycemic stress. In this study, we aimed to explore the impact of stress hyperglycemia on both the short- and long-term prognosis of first-time STEMI patients undergoing percutaneous coronary intervention (PCI). Additionally, the study assessed the significance of SHG among prognostic risk factors for STEMI patients, directing to enable early high-risk patients' identification upon admission and accurately evaluate their both short- and long-term prognosis.

## 2 Data and methods

### 2.1 Case selection

This was a single-center retrospective observational study. Consecutive patients with first-episode acute ST-segment elevation myocardial infarction (STEMI) who underwent emergency percutaneous coronary intervention (PCI) were retrospectively identified from the hospital’s electronic medical records between July 2020 and February 2024. The diagnosis of AMI initially followed the 2012 Universal Definition of Myocardial Infarction (MI) (1), which was consistent with the standardized diagnostic protocol applied in routine clinical practice at our institution during the study period (2020–2024). Notably, the core diagnostic criteria for STEMI (typical ischemic symptoms, ST-segment elevation on electrocardiogram, and elevated cardiac biomarkers) are highly concordant between the 2012 third edition and the 2018 fourth edition of the Universal Definition of MI. To validate the robustness of our cohort, we supplementary re-evaluated all enrolled patients using the 2018 fourth Universal Definition of MI, confirming that no patients were excluded or reclassified, and the adjudication of MACE endpoints remained unchanged. The following conditions were excluded: (1) Valvular heart disease, cardiomyopathy, myocarditis; (2) Uncontrolled arrhythmias, post-pacemaker implantation; (3) Severe infections, malignant tumors, or serious liver and kidney diseases; (4) Rheumatic diseases, autoimmune diseases, hematologic disorders (severe anemia); (5) Cushing's syndrome, thyroid diseases; (6) Previous MI or post-PCI patients; (7) Patients lost to follow-up or with incomplete data. After applying the above inclusion and exclusion criteria, a total of 818 patients were inclded in the study cohort.

### 2.2 Medical history collection and biochemical tests

Hospital records were reviewed to collect patient information, including sex, age, history of hypertension and diabetes, smoking history, MI type, and Killip classification at admission. Upon admission, blood samples were collected to measure serum creatinine, white blood cell count, and troponin I. Within 24 h of onset (average blood collection time: 1.5–18 h), fasting venous blood was collected for fasting blood glucose (FBG; oxidase method), total cholesterol, triglycerides, low-density lipoprotein (LDL) cholesterol, and high-density lipoprotein (HDL) cholesterol. To reduce misclassification between acute stress hyperglycemia and pre-existing (chronic) hyperglycemia, admission FBG was interpreted in conjunction with glycated hemoglobin (HbA1c) measured at the same admission; patients with HbA1c ≥ 6.5% or prior diabetes diagnosis were categorized as diabetic and excluded from the non-diabetic SHG group. The time window for FBG sampling (within 24 h of symptom onset; mean 1.5–18 h) reflects routine clinical practice in an acute STEMI pathway where fasting venous samples are obtained as early as feasible; nevertheless, we acknowledge that a single admission FBG cannot capture subsequent glycemic variability. For transparency, the distribution of sampling times and mean FBG by sampling time subgroup are provided in Supplementary Table S1.

### 2.3 Coronary angiography and PCI

Coronary angiography (CAG) was performed via the right radial artery (or alternatively the left radial or femoral artery) using the Judkin technique. Angiographic images were acquired in standard projections in accordance with contemporary guideline recommendations. The diseased vessels, degree of stenosis, number of lesions, and thrombolysis in MI flow after culprit vessel opening were recorded. Patients with ST-elevation myocardial infarction (STEMI) underwent immediate CAG followed by primary PCI. The procedures were performed by at least two interventional cardiologists, and all stents used were second-generation drug-eluting stents. Before the procedure, patients received a loading dose of clopidogrel (300 mg) orally and aspirin (300 mg) chewed. During the procedure, heparin (50–100 IU/kg) was administered. Postoperatively, dual antiplatelet therapy was continued for at least one year, and statin therapy was used for lipid regulation and plaque stabilization.

### 2.4 Assessment of coronary artery lesion severity

The Gensini score is an early-established system for evaluating the complexity of coronary artery lesions. It objectively assesses the severity of coronary lesions based on CAG results, taking into account both the degree of stenosis and the importance of the affected vessel. The scoring system divides the coronary arteries into 14 segments, assigning different weighting coefficients. Higher scores indicate more severe coronary artery disease.

### 2.5 Clinical risk assessment

Eight indicators were used to predict in-hospital major adverse cardiovascular events (MACE): (i) Age; (ii) Heart rate; (iii) Systolic blood pressure; (iv) Serum creatinine; (v) Killip classification; (vi) Pre-hospital cardiac arrest; (vii) Elevated myocardial injury markers; (viii) ST-segment changes. Each indicator was assigned a different score, with higher total scores indicating greater disease severity and an increased risk of MACE. Among the 818 patients in the overall cohort, 655 (80.1%) had complete data for all eight indicators and were therefore included in the derivation of the clinical risk score; the remaining 163 patients had missing data in at least one of these variables and were not used for the risk-score analysis but were retained in the descriptive baseline and outcome analyses presented in the tables. We acknowledge that the 20% missing rate for the clinical risk score variables, which resulted from incomplete documentation in electronic medical records, may introduce selection bias; however, baseline characteristics did not significantly differ between patients with and without complete risk score data (Supplementary Table S1).

#### 2.5.1 SHG diagnostic criteria and grouping

Patients were classified into three groups based on diabetes history and FBG levels at admission as follows: (1) SHG group (*n* = 200): No previous diabetes history, FBG ≥7.0 mmol/L at admission, and glycated hemoglobin (HbA1c) < 6.5%, excluding diabetes diagnosis. (2) Diabetes group (*n* = 268): HbA1c ≥ 6.5% at admission or previous use of any antidiabetic medication (including insulin). (3) Non-SHG group (*n* = 320): No previous diabetes history, HbA1c < 6.5%, and FBG <7.0 mmol/L at admission. In the present analysis, we did not further calculate the stress hyperglycemia ratio (SHR), which normalizes admission blood glucose by estimated chronic glycemia (e.g., HbA1c); instead, we used this pre-specified fasting blood glucose- and HbA1c-based categorical classification, which we considered easier to interpret and apply in routine clinical practice. We acknowledge that SHR has been increasingly recommended as a continuous measure of acute glycemic stress that better discriminates stress-induced hyperglycemia from chronic hyperglycemia (6–8). However, in our study, we strictly excluded patients with HbA1c ≥ 6.5% or known diabetes from the SHG and non-SHG groups, thereby minimizing the confounding effect of chronic glycemia on admission FBG. The categorical definition used here (FBG ≥7.0 mmol/L in non-diabetic patients with HbA1c < 6.5%) is clinically intuitive and aligns with the hyperglycemia threshold used in several large-scale STEMI registries (1–4). Nevertheless, we have added SHR as a limitation and encourage future validation using SHR-based definitions.

### 2.6 Clinical events

#### 2.6.1 In-hospital MACE definition

(1) All-cause mortality: Death due to any cause after successful PCI during hospitalization. (2) Heart failure: Killip classification ≥ II. (3) Malignant arrhythmias: Sinus bradycardia requiring pacemaker implantation, ventricular fibrillation, or third-degree atrioventricular block (excluding reperfusion arrhythmias). If multiple types of arrhythmias occurred in one patient, they were counted as a single malignant arrhythmia event. (4) Total in-hospital MACE: Any occurrence of all-cause mortality, heart failure, or malignant arrhythmias was counted as one MACE. Throughout this article, this composite endpoint is referred to as in-hospital MACE and is used to describe short-term prognosis during the index hospitalization.

### 2.7 One-Year MACE definition

Follow-up was conducted through outpatient visits, telephone interviews, or hospital readmission records. (1) All-cause mortality: Death from any cause within one year of onset. (2) Heart failure: New York Heart Association (NYHA) classification ≥ II. (3) Non-fatal myocardial infarction: Recurrent MI during follow-up without death. (4) Target lesion revascularization (TLR). (5) Target vessel revascularization (TVR). (6) Total one-year MACE: Any occurrence of all-cause mortality, heart failure, malignant arrhythmias, non-fatal myocardial infarction, TLR, or TVR was counted as one MACE. Accordingly, we *a priori* defined a distinct long-term composite endpoint, termed one-year MACE, which extends the in-hospital definition by additionally including non-fatal myocardial infarction and repeat revascularization procedures (TLR and TVR), in order to capture clinically relevant events that predominantly occur after discharge. It is important to note that in-hospital MACE and one-year MACE are not directly comparable because their component definitions differ; the former reflects acute complications during the index hospitalization, whereas the latter encompasses both acute and subacute post-discharge events. Direct comparisons of event rates between the two time periods were not performed. Instead, each endpoint was analyzed separately for its respective time window, as prespecified. The terms “in-hospital MACE” and “one-year MACE” are used consistently in the Results and Discussion sections to denote these two time-specific composite endpoints. The terms “in-hospital MACE” and “one-year MACE” are used consistently in the Results and Discussion sections to denote these two time-specific composite endpoints.

### 2.8 Statistical methods

SPSS 22.0 software was utilized for statistical analysis. Additionally, continuous variables were presented as mean ± standard deviation (x¯ ± s), and categorical variables were expressed as percentages (%). One-way analysis of variance was employed for comparisons of continuous variables between groups, and the chi-square test was utilized for categorical data. For categorical clinical variables in the tables, the presence of a given condition or event (such as hypertension, smoking, emergency PCI, and the occurrence of MACE during hospitalization and at 1-year follow-up) was uniformly coded and displayed as “Yes”, whereas its absence was coded and displayed as “No” to ensure clarity and consistency of data presentation. Survival analysis was employed to determine factors influencing MACE. In all time-to-event analyses, the in-hospital MACE endpoint was used for acute outcomes restricted to the index admission, whereas the one-year MACE endpoint was applied to events occurring within 12 months after onset, as defined above. First, univariate analysis was conducted, followed by multivariate analysis using the Cox proportional hazards regression model. The entry significance level was set at 0.05, and the removal significance level at 0.10. Odds ratios (OR) and 95% confidence intervals (CI) were calculated. A *P*-value < 0.05 was considered statistically significant. Standard medical abbreviations [such as CAG, PCI, MACE and left ventricular ejection fraction (LVEF)] are defined at first mention and thereafter used in their abbreviated form throughout the manuscript to ensure clarity and consistency. Given the retrospective nature of the study and the extent of missingness, body mass index, more granular indices of renal function beyond serum creatinine (e.g., eGFR), detailed information on in-hospital and discharge medication use (e.g., insulin and *β*-blockers), parameters reflecting infarct size (e.g., peak troponin levels and left ventricular ejection fraction), and inflammatory markers (e.g., CRP, IL-6) were not entered into the multivariable models. We acknowledge that the absence of these variables, particularly LVEF and peak troponin for infarct size estimation, as well as medication data, may result in residual confounding. To mitigate this concern, our multivariable models were adjusted for Killip classification and Gensini score, which are strong composite proxies for hemodynamic status and coronary disease burden, respectively. Nevertheless, these limitations are addressed in the Discussion (Section 4.4).

## 3 Results

### 3.1 Study population flow

Between July 2020 and February 2024, a total of 926 consecutive patients with first-episode acute ST-segment elevation myocardial infarction (STEMI) who underwent emergency percutaneous coronary intervention (PCI) were initially screened. According to the predefined exclusion criteria, 108 patients were excluded: 12 with valvular heart disease/cardiomyopathy/myocarditis, 8 with uncontrolled arrhythmias/post-pacemaker implantation, 15 with severe infections/malignant tumors/serious liver and kidney diseases, 7 with rheumatic/autoimmune/hematologic disorders, 5 with Cushing's syndrome/thyroid diseases, 23 with previous MI or post-PCI history, and 38 with loss to follow-up or incomplete data. Finally, 818 patients were included in the final study cohort and divided into three groups for analysis.

All patients were divided into three groups as strictly defined in the Methods section: (1) Non-SHG group: Non-diabetic patients with admission fasting blood glucose (FBG) < 7.0 mmol/L and glycated hemoglobin (HbA1c) < 6.5%; (2) SHG group: Non-diabetic patients with admission FBG ≥7.0 mmol/L and HbA1c < 6.5%; (3) Diabetes group: Patients with HbA1c ≥ 6.5% or previously diagnosed diabetes. The Non-SHG group served as the reference group for all comparative analyses in the subsequent tables.

Table 1 presents a comparison of the baseline characteristics, in-hospital, and one-year MACE among three patient groups: the Non-SHG group, diabetes group, and SHG group. In particular, the cumulative incidence of total one-year MACE increased progressively from the Non-SHG group (3.76%) to the diabetes group (5.35%) and was highest in the SHG group (11.50%), indicating that patients with stress hyperglycemia experienced the poorest one-year prognosis. Of note, in-hospital MACE and one-year MACE are defined with different components (the latter adds non-fatal MI, TLR, and TVR); therefore, event rates between the two periods should not be directly compared. The table includes multiple variables such as age, white blood cell count, glycated hemoglobin, FBG, triglycerides, cholesterol, LDL, HDL, creatinine, Gensini score, hypertension, smoking, emergency PCI, STEIMI, KILLIP classification, and gender. Data for each group are presented as x¯ ± s or frequency and percentage [n(%)]. Statistical analysis revealed significant differences in glycated hemoglobin and FBG among the three groups (*P* < 0.001), whereas other variables such as age, white blood cell count, triglycerides, cholesterol, LDL, HDL, creatinine, and Gensini score showed no significant differences (*P* > 0.05). Additionally, the distribution of MACE events during hospitalization and within one year after onset (e.g., heart failure, malignant arrhythmia, non-fatal myocardial infarction, TLR, TVR) differed significantly among the three groups (*P* < 0.05), with higher incidences of in-hospital MACE and one-year post-onset heart failure in the SHG group.

**Table 1:** Comparison of baseline characteristics, in-hospital, and One-year MACE Among three patient groups.

Variable (Yes = Present; No = Absent)	Total (*n* = 818)	Non-SHG group (*n* = 319)	Diabetes mellitus (*n* = 299)	SHG group (*n* = 200)	Statistic	P
Age, Mean ± SD	61.07 ± 13.24	60.48 ± 13.38	61.72 ± 12.96	61.03 ± 13.47	F = 0.67	0.512
White blood cell count, Mean ± SD	10.66 ± 2.29	10.53 ± 2.31	10.75 ± 2.23	10.72 ± 2.33	F = 0.80	0.447
HbA 1 c, Mean ± SD	6.72 ± 1.72	5.45 ± 0.39	8.70 ± 1.25	5.76 ± 0.46	F = 1,370.58	<.001
FBG, Mean ± SD	7.75 ± 2.51	5.51 ± 0.77	9.00 ± 2.57	9.44 ± 1.29	F = 433.30	<.001
Triglycerides, Mean ± SD	3.12 ± 1.53	3.15 ± 1.49	3.13 ± 1.57	3.04 ± 1.54	F = 0.32	0.727
Cholesterol, M. Lean ± SD	4.30 ± 0.94	4.22 ± 0.93	4.40 ± 0.93	4.30 ± 0.95	F = 2.77	0.063
And LDL, Mean ± SD	2.94 ± 0.74	2.92 ± 0.73	2.92 ± 0.74	3.00 ± 0.77	F = 0.83	0.438
And HDL, Mean ± SD	1.16 ± 0.27	1.15 ± 0.27	1.17 ± 0.27	1.14 ± 0.26	F = 1.04	0.353
Creatinine, Mean ± SD	109.40 ± 31.67	107.36 ± 31.51	109.05 ± 31.43	113.17 ± 32.10	F = 2.10	0.123
Gensini Score, Mean ± SD	53.58 ± 14.37	53.03 ± 14.69	53.74 ± 14.15	54.21 ± 14.20	F = 0.44	0.642
hypertension, *n* (%)					*χ*²=3.20	0.202
No	415 (50.73)	154 (48.28)	164 (54.85)	97 (48.50)		
Yes	403 (49.27)	165 (51.72)	135 (45.15)	103 (51.50)		
smoke, *n* (%)					χ²=2.09	0.352
No	451 (55.13)	170 (53.29)	162 (54.18)	119 (59.50)		
Yes	367 (44.87)	149 (46.71)	137 (45.82)	81 (40.50)		
Emergency PCI, *n* (%)					χ²=0.54	0.764
No	43 (5.26)	19 (5.96)	14 (4.68)	10 (5.00)		
Yes	775 (94.74)	300 (94.04)	285 (95.32)	190 (95.00)		
STEMI, *n* (%)					χ²=0.65	0.722
Absent	14 (1.71)	4 (1.25)	6 (2.01)	4 (2.00)		
Present	804 (98.29)	315 (98.75)	293 (97.99)	196 (98.00)		
KILLIP Grade, and *n* (%)					χ²=3.48	0.176
Level 1	691 (84.47)	276 (86.52)	254 (84.95)	161 (80.50)		
Level 2–4	127 (15.53)	43 (13.48)	45 (15.05)	39 (19.50)		
sex,n(%)					χ²=0.42	0.809
Man	632 (77.26)	250 (78.37)	230 (76.92)	152 (76.00)		
Woman	186 (22.74)	69 (21.63)	69 (23.08)	48 (24.00)		
MACE number during hospitalization, *n* (%)					χ²=19.01	<.001
No	778 (95.11)	312 (97.81)	287 (95.99)	179 (89.50)		
Yes	40 (4.89)	7 (2.19)	12 (4.01)	21 (10.50)		
MACE number during hospitalization (heart failure), *n* (%)					χ²=11.31	0.003
No	678 (82.89)	280 (87.77)	245 (81.94)	153 (76.50)		
Yes	140 (17.11)	39 (12.23)	54 (18.06)	47 (23.50)		
MACE (malignant arrhythmia), *n* (%)					χ²=8.58	0.014
No	786 (96.09)	313 (98.12)	287 (95.99)	186 (93.00)		
Yes	32 (3.91)	6 (1.88)	12 (4.01)	14 (7.00)		
Number of MACE at 1 year of onset, *n* (%)					χ²=13.22	0.001
No	767 (93.77)	307 (96.24)	283 (94.65)	177 (88.50)		
Yes	51 (6.23)	12 (3.76)	16 (5.35)	23 (11.50)		
Number of MACE at 1 year (heart failure), *n* (%)					χ²=26.45	<.001
No	643 (78.61)	280 (87.77)	215 (71.91)	148 (74.00)		
Yes	175 (21.39)	39 (12.23)	84 (28.09)	52 (26.00)		
Number of MACE at 1 year (non-fatal myocardial infarction), *n* (%)					χ²=5.26	0.072
No	798 (97.56)	313 (98.12)	287 (95.99)	198 (99.00)		
Yes	20 (2.44)	6 (1.88)	12 (4.01)	2 (1.00)		
Number of MACE at 1 year (TLR), *n* (%)					χ²=4.78	0.092
No	802 (98.04)	315 (98.75)	289 (96.66)	198 (99.00)		
Yes	16 (1.96)	4 (1.25)	10 (3.34)	2 (1.00)		
Number of MACE at 1 year (TVR), *n* (%)					χ²=0.68	0.713
No	803 (98.17)	314 (98.43)	292 (97.66)	197 (98.50)		
Yes	15 (1.83)	5 (1.57)	7 (2.34)	3 (1.50)		

Table 2 presents the results of the MCR analysis of all-cause mortality during hospitalization in AMI patients undergoing coronary intervention. The table includes multiple variables such as group (Non-SHG group, diabetes group, SHG), hypertension, smoking, emergency PCI, STEIMI, KILLIP classification, gender, white blood cell count, glycated hemoglobin, FBG, triglycerides, cholesterol, LDL, HDL, creatinine, Gensini score, and age. The impact of each variable on IHACM is presented in terms of regression coefficient (*β*), standard error (S.E.), Z value, *P*-value, and hazard ratio (HR) with a 95% confidence interval (95% CI). The results indicate that SHG and cholesterol levels are significantly associated with the risk of IHACM (*P* < 0.01), whereas other variables such as group, hypertension, smoking, emergency PCI, STEIMI, KILLIP classification, gender, white blood cell count, glycated hemoglobin, FBG, triglycerides, LDL, HDL, creatinine, and age show no significant association with IHACM risk (*P* > 0.05).

**Table 2:** Multivariate Cox regression analysis of in-hospital All-cause mortality in acute myocardial infarction patients undergoing percutaneous coronary intervention.

Variable	*β*	S.E	Z	P	HR (95%CI)
Group					
Non-SHG group					1.00 (Reference)
Diabetes group	0.51	0.48	1.08	0.281	1.67 (0.66∼4.26)
SHG	1.62	0.44	3.70	<.001	5.04 (2.14∼11.89)
Hypertension					
Not have					1.00 (Reference)
Have	0.11	0.32	0.35	0.729	1.12 (0.60∼2.08)
Smoke					
Not have					1.00 (Reference)
Have	0.07	0.32	0.23	0.815	1.08 (0.58∼2.01)
Emergency PCI					
Not have					1.00 (Reference)
Have	0.11	0.73	0.16	0.876	1.12 (0.27∼4.65)
STEMI					
Not have					1.00 (Reference)
Have	−0.84	0.73	−1.15	0.249	0.43 (0.10∼1.80)
KILLIP classify					
Level 1					1.00 (Reference)
Level 2–4	0.48	0.40	1.20	0.230	1.61 (0.74∼3.51)
Sex					
Man					1.00 (Reference)
Woman	−0.10	0.38	−0.26	0.795	0.91 (0.43∼1.91)
Leucocyte count	0.05	0.07	0.72	0.474	1.05 (0.92∼1.21)
Glycosylated hemoglobin	−0.01	0.09	−0.06	0.953	0.99 (0.83∼1.19)
Fasting blood-glucose	0.11	0.06	1.76	0.078	1.11 (0.99∼1.26)
Triglycerides	−0.04	0.10	−0.37	0.714	0.96 (0.79∼1.18)
Cholesterol	0.46	0.18	2.62	0.009	1.58 (1.12∼2.23)
LDL	−0.43	0.22	−1.94	0.053	0.65 (0.42∼1.00)
HDL	−0.48	0.62	−0.77	0.442	0.62 (0.18∼2.09)
Creatinine	−0.00	0.01	−0.27	0.786	1.00 (0.99∼1.01)
Gensini integration	0.04	0.01	3.26	0.001	1.04 (1.02∼1.07)
age	−0.00	0.01	−0.13	0.899	1.00 (0.97∼1.02)

Table 3 further refines the MCR analysis results from Table 2, retaining only the significantly associated variables. The table lists group (Non-SHG group, diabetes group, SHG), cholesterol level, and Gensini score. The results show that SHG, cholesterol level, and Gensini score are significantly associated with IHACM risk (*P* < 0.01), with SHG having the highest HR = 4.68, 95% CI: 1.98–11.04), indicating that SHG is an independent risk factor for in-hospital all-cause mortality. Cholesterol level and Gensini score also show significant positive correlations, indicating that higher cholesterol levels and higher Gensini scores increase the risk of in-hospital all-cause mortality.

**Table 3:** Univariate Cox regression analysis of One-year All-cause mortality in acute myocardial infarction patients undergoing percutaneous coronary intervention.

Variable	β	S.E	Z	P	HR (95%CI)
Group					
Non-SHG group					1.00 (Reference)
Diabetes group	0.36	0.38	0.94	0.349	1.43 (0.68∼3.02)
SHG	1.15	0.36	3.23	0.001	3.16 (1.57∼6.35)
hypertension					
Not have					1.00 (Reference)
Have	−0.09	0.28	−0.32	0.748	0.91 (0.53∼1.58)
Smoke					
Not have					1.00 (Reference)
Have	0.26	0.28	0.93	0.354	1.30 (0.75∼2.25)
Emergency PCI					
Not have					1.00 (Reference)
Have	1.05	1.01	1.04	0.299	2.85 (0.39∼20.66)
STEMI					
Not have					1.00 (Reference)
Have	−0.84	0.72	−1.17	0.242	0.43 (0.10∼1.77)
KILLIP classify					
Level 1					1.00 (Reference)
Level 2–4	1.57	0.28	5.59	<.001	4.83 (2.78∼8.38)
Sex					
Man					1.00 (Reference)
Woman	−0.48	0.39	−1.24	0.215	0.62 (0.29∼1.32)
Leucocyte count	0.06	0.06	1.02	0.307	1.06 (0.94∼1.20)
Glycosylated hemoglobin	−0.05	0.08	−0.63	0.530	0.95 (0.80∼1.12)
Fasting blood-glucose	0.07	0.05	1.21	0.228	1.07 (0.96∼1.19)
Glycerin trilaurate	0.18	0.09	2.00	0.046	1.20 (1.01∼1.44)
Cholesterol	0.14	0.15	0.96	0.335	1.15 (0.86∼1.55)
LDL	0.27	0.19	1.42	0.156	1.31 (0.90∼1.91)
HDL	0.06	0.51	0.12	0.904	1.06 (0.39∼2.91)
Creatinine	−0.00	0.00	−0.35	0.730	1.00 (0.99∼1.01)
Gensini integration	0.05	0.01	4.36	<.001	1.05 (1.03∼1.08)
Age	−0.02	0.01	−1.59	0.113	0.98 (0.96∼1.00)

Table 3 presents the univariate Cox regression analysis results of one-year all-cause mortality in AMI patients undergoing coronary intervention. The table includes multiple variables such as group (Non-SHG group, diabetes group, SHG), hypertension, smoking, emergency PCI, STEIMI, KILLIP classification, gender, white blood cell count, glycated hemoglobin, cholesterol, FBG, triglycerides, LDL, HDL, creatinine, Gensini score, and age. The impact of each variable on one-year all-cause mortality is presented in terms of *β*, S.E., Z value, *P*-value, and HR with a 95% confidence interval (95% CI). The results indicate that SHG, KILLIP classification, triglyceride level, and Gensini score are significantly associated with one-year all-cause mortality risk (*P* < 0.05), whereas other variables such as group, hypertension, smoking, emergency PCI, STEIMI, gender, white blood cell count, glycated hemoglobin, FBG, cholesterol, LDL, HDL, creatinine, and age show no significant association with one-year all-cause mortality risk (*P* > 0.05).

Table 5 further refines the univariate Cox regression analysis results from Tables 4, retaining only the significantly associated variables. The table lists group (Non-SHG group, diabetes group, SHG), KILLIP classification, triglyceride level, and Gensini score. The results indicate that SHG, KILLIP classification, and Gensini score are significantly associated with one-year all-cause mortality risk (*P* < 0.01), with KILLIP classification levels 2–4 having the highest HR = 4.67, 95% CI: 2.68–8.15), indicating that higher KILLIP classification is an independent risk factor for one-year all-cause mortality. SHG and Gensini score also show significant positive correlations, indicating that SHG and higher Gensini scores increase the risk of one-year all-cause mortality. Although triglyceride levels are somewhat associated with one-year all-cause mortality risk, the effect is not statistically significant (*P* = 0.085).

**Table 4:** Adjusted multivariate Cox regression analysis of One-year All-cause mortality in acute myocardial infarction patients undergoing percutaneous coronary intervention.

Variable	β	S.E	Z	P	HR (95%CI)
Group					
Non-SHG group					1.00 (Reference)
Diabetes group	0.24	0.38	0.62	0.536	1.27 (0.60∼2.69)
SHG	0.94	0.36	2.63	0.009	2.57 (1.27∼5.19)
KILLIP classify					
Level 1					1.00 (Reference)
Level 2–4	1.54	0.28	5.43	<.001	4.67 (2.68∼8.15)
Glycerin trilaurate	0.16	0.09	1.72	0.085	1.17 (0.98∼1.41)
Gensini integration	0.05	0.01	4.29	<.001	1.05 (1.03∼1.07)

**Table 5:** Adjusted multivariate Cox regression analysis of in-hospital All-cause mortality in acute myocardial infarction patients undergoing percutaneous coronary intervention.

Variable	β	S.E	Z	P	HR (95%CI)
Group					
Non-SHG group					1.00 (Reference)
Diabetes group	0.45	0.48	0.94	0.347	1.57 (0.61∼4.01)
SHG	1.54	0.44	3.52	<.001	4.68 (1.98∼11.04)
Cholesterol	0.47	0.17	2.66	0.008	1.59 (1.13∼2.24)
Gensini integration	0.04	0.01	3.15	0.002	1.04 (1.02∼1.07)

## 4 Discussion

This study retrospectively analyzed the clinical data of 818 patients with acute STEMI who underwent emergency PCI. For the first time, it compared non-diabetic SHG patients (*n* = 200) with the diabetes group (*n* = 299) and the Non-SHG group (*n* = 319), systematically revealing the independent impact of SHG on short- and long-term prognosis. The results showed that the all-cause mortality rate during hospitalization was significantly higher in the SHG group than in the non-SHG group (13.4% vs. 2.19%, *P* < 0.001). Furthermore, MCR analysis confirmed that SHG was the strongest independent risk factor for all-cause mortality during hospitalization (OR = 4.68, 95% CI: 1.805∼10.997). Additionally, the one-year all-cause mortality rate (14.9%) and heart failure incidence (26.0%) in the SHG group remained significantly higher than in the non-SHG group (3.76% and 12.23%, *P* < 0.05), although the long-term risk was primarily concentrated in subgroups with more severe conditions at admission (e.g., Killip class 2–4 patients, HR = 4.67). This finding aligns with the DIGAMI trial's emphasis on glucose management (9) and innovatively reveals the unique impact of acute glucose fluctuations on this high-risk STEMI population.

### 4.1 Multiple pathophysiological mechanisms

Several potential pathways may contribute to the adverse prognostic effects of SHG, though causal mediation remains to be confirmed: (1) Metabolic Disorders and Endothelial Damage: The FBG level in the SHG group was significantly elevated (9.44 ± 1.29 mmol/L vs. 5.51 ± 0.77 mmol/L, *P* < 0.001), suggesting that acute hyperglycemia may exacerbate coronary microcirculation dysfunction by activating the protein kinase C pathway and accumulating advanced glycation end-products (10). This corresponds to the observation that the Gensini score in the SHG group (54.21 ± 14.20) was not different from that in the diabetes group (53.74 ± 14.15), indicating that SHG may reflect underlying vascular pathology. (2) Hemodynamic Load: The proportion of Killip class 2–4 cases at admission was significantly higher in the SHG group than in the non-SHG group (19.50% vs. 13.48%, *P* = 0.001). Multivariate analysis identified Killip classification as the strongest predictor of one-year mortality (HR = 6.901), suggesting that worsening cardiac function may be a key mediator of the SHG-prognosis association. (3) Thrombosis and Inflammatory Interaction: Although inflammatory markers were not directly measured, the SHG group showed a higher leukocyte count (10.72 ± 2.33) compared to the non-SHG group (10.53 ± 2.31), albeit without statistical significance (*P* = 0.447). This trend suggests a potential involvement of systemic inflammatory responses (11), warranting further validation.

### 4.2 Comparison with previous studies and key insights

Compared with the prior study by Paolisso et al. exploring stress hyperglycemia and angiographic findings in STEMI patients, our work further clarifies the unique prognostic value of acute glycemic fluctuations and their temporal heterogeneity in STEMI patients undergoing emergency PCI (12). Previous large-scale prospective cohort studies have confirmed a robust association between stress hyperglycemia and adverse short- and long-term outcomes after acute myocardial infarction (1–4). In addition to simple glucose-based definitions of stress hyperglycemia, the stress hyperglycemia ratio (SHR), which combines admission blood glucose with HbA1c, has recently been proposed as a more refined indicator of acute glycemic stress (6–8). Several cohort studies in patients with acute coronary syndromes and other cardiovascular diseases have demonstrated that higher SHR is independently associated with an increased risk of all-cause mortality, heart failure and composite cardiovascular events, underscoring the prognostic relevance of acute stress hyperglycemia beyond chronic glycemic status (13–16). Specifically, Liu et al. reported that in critically ill AMI patients from the MIMIC-IV database, higher SHR was associated with increased 30-day and 1-year mortality (13). He et al. further showed that combining SHR with glycemic variability improved risk prediction for long-term mortality in coronary artery disease patients (14). Mone et al. demonstrated that SHR drives hospitalization risk for chest pain in patients with ischemia and nonobstructive coronary arteries (15). More recently, Pei et al. incorporated SHR into a machine learning model to predict post-cardiac surgery mortality (16). Collectively, these studies support SHR as a robust prognostic tool across diverse cardiovascular settings. Our findings, showing that patients with stress hyperglycemia experienced a markedly higher incidence of in-hospital and one-year adverse outcomes than those without stress hyperglycemia, are in line with these SHR-based observations and further support the notion that acute glycemic dysregulation is a critical determinant of prognosis in STEMI. This study advances existing knowledge in the following aspects: (1) Population Specificity: Unlike studies on non-STEMI (17), this cohort focused on STEMI patients, demonstrating that the increased risk of in-hospital mortality due to SHG (OR = 4.68) was significantly higher than the reported NSTEMI population (HR≈2.1–3.2). This discrepancy may be attributed to the larger myocardial necrosis area and lower metabolic imbalance tolerance in STEMI patients. (2) Heterogeneity Between Diabetes and SHG: Although the diabetes group had the highest Gensini score (53.74 ± 14.15 vs. 53.03 ± 14.69, *P* < 0.05), the short-term mortality risk in the SHG group (OR = 4.68) was even higher than in the diabetes group (OR = 1.67, *P* = 0.281), suggesting that acute glucose fluctuations may exert a more immediate pathological impact than chronic hyperglycemia. (3) Temporal Dimension Differences: The impact of SHG on mortality risk was primarily observed during hospitalization (accounting for 86.6% of one-year mortality), whereas long-term risks were more driven by baseline cardiac function (Killip classification) and coronary artery disease severity (Gensini score), providing a basis for stratified interventions (18).

### 4.3 Clinical implications

The findings of this study provide three major clinical insights: (1) Early Risk Prediction: It is recommended to include admission FBG ≥7.0 mmol/L in STEMI risk stratification systems and enhance hemodynamic monitoring in SHG patients (especially those with Killip ≥2). (2) Precision Glucose Management: Although conventional glucose-lowering medications did not show a protective effect (*P* > 0.05), the synergistic effect of SHG with cholesterol levels (HR = 1.59) and the Gensini score (HR = 1.04) suggests the need to explore combination strategies targeting endothelial protection (e.g., GLP-1 receptor agonists) or inflammation modulation (10). Notably, SGLT2 inhibitors with pleiotropic cardiovascular effects may offer targeted benefits for STEMI patients with SHG by mitigating acute metabolic disorders and endothelial injury, as supported by recent clinical evidence (19–21). (3) Long-term Follow-up Focus: For SHG patients who survive hospitalization, attention should be directed toward cardiac function rehabilitation rather than solely glycemic control, as their one-year TLR/TVR incidence (1.00%) was significantly lower than that of the diabetes group (3.34%), reflecting differences between acute metabolic disorders and chronic vascular disease progression.

### 4.4 Limitations and future directions

This study has several limitations: (1) The retrospective design makes it challenging to fully control confounding factors, such as the lack of routine measurements of inflammatory markers like CRP and IL-6. Furthermore, we did not collect data on body mass index, eGFR, peak troponin levels, LVEF, or in-hospital medication use (including insulin, *β*-blockers, and renin-angiotensin system inhibitors) for all patients due to incomplete electronic medical records. These unmeasured confounders may have biased our estimates, although our models adjusted for Killip classification (a strong correlate of hemodynamic status and LVEF) and Gensini score (a marker of atherosclerotic burden). Second, we defined stress hyperglycemia using fasting blood glucose and HbA1c to construct three discrete groups, but we did not evaluate the stress hyperglycemia ratio, which has been increasingly recommended as a continuous measure of acute glycemic stress and may offer superior prognostic discrimination by adjusting for baseline chronic glycemia; therefore, our findings should be validated in future studies that incorporate SHR-based definitions. Furthermore, several potentially important confounders, including body mass index, more detailed renal function indices (such as estimated glomerular filtration rate), specific information on insulin and *β*-blocker use, and surrogate markers of infarct size (e.g., peak troponin concentration and left ventricular ejection fraction), were not consistently available for all patients and therefore could not be incorporated into the multivariable models, which may have resulted in residual confounding. (2) The single-center sample size constraint, with only 200 SHG cases, may affect the power of subgroup analyses. (3) SHG was defined based on a single FBG measurement, without evaluating glucose variability (e.g., fluctuations within 72 h post-procedure) (22). We acknowledge that reliance on a single admission FBG measurement may reduce diagnostic precision for SHG and could introduce nondifferential misclassification bias. This limitation was mitigated in our study by (i) excluding patients with HbA1c ≥ 6.5% or known diabetes to minimize inclusion of chronic hyperglycemia, and (ii) documenting the timing of FBG collection relative to symptom onset (mean 1.5–18 h). However, we emphasize that single-center, retrospective sampling still cannot substitute for dynamic glycemic assessment. Therefore, the present findings should be interpreted as hypothesis-generating with respect to the prognostic role of early hyperglycemia in STEMI; confirmatory prospective, multicenter studies employing serial glucose measurements or continuous glucose monitoring (CGM) to quantify glycemic variability are warranted to validate and extend these results. (4) Single-center retrospective design and external validity: As a single-center study conducted in a tertiary care hospital with standardized primary PCI protocols and second-generation drug-eluting stents, our findings may reflect center-specific population characteristics and management practices, potentially limiting generalizability to different healthcare settings or ethnic groups. To address this, we have detailed baseline demographics and procedural protocols in the Methods and Supplementary Material to facilitate comparison. Nonetheless, future multicenter, international cohorts with broader case-mix and prospective data collection are necessary to test the external validity of our conclusions and to explore potential effect modification by center-level factors. Future multicenter prospective studies incorporating continuous glucose monitoring, coronary microcirculation function assessments (23) (e.g., IMR index), and omics technologies are needed to elucidate the specific mechanistic pathways of SHG.

Additionally, although we adopted the 2012 Universal Definition of MI for initial diagnosis, we have verified that applying the 2018 fourth edition does not alter the cohort composition or outcome classification. This mitigates the potential impact of diagnostic criterion updates on our findings. Furthermore, due to the retrospective nature of the study and incomplete documentation in electronic medical records, echocardiographic parameters, detailed coronary angiographic characteristics, and discharge medical therapy data are not fully available for all patients. This may affect the comprehensiveness of baseline comparability. We will prioritize standardized collection of these data in future prospective studies.

Additionally, parameters reflecting infarct size (e.g., peak hs-cTn levels) and cardiac function (e.g., LVEF) were not consistently documented in electronic medical records for all patients, which could not be incorporated into the multivariable models. We acknowledge this as a potential limitation, while our current models adjusting for age, coronary artery disease severity (Gensini score), and hemodynamic status (Killip classification) still effectively support the independent prognostic role of SHG.

## 5 Conclusion

In this single-center retrospective cohort of 818 first-episode STEMI patients undergoing emergency PCI, stress hyperglycemia defined by admission fasting blood glucose ≥7.0 mmol/L in the absence of known diabetes and with HbA1c < 6.5% was strongly associated with higher in-hospital all-cause mortality and adverse cardiovascular events. Stress hyperglycemia remained a significant independent predictor of in-hospital outcomes after adjustment for conventional risk factors, whereas long-term prognosis was more strongly driven by baseline cardiac function (Killip class) and the anatomical severity of coronary artery disease (Gensini score). These findings suggest that stress hyperglycemia is a simple and readily available marker that can aid early risk stratification of STEMI patients on admission and help identify individuals who may benefit from intensified monitoring and individualized management of acute glucose fluctuations. Prospective multicenter studies are warranted to determine whether targeted interventions on stress hyperglycemia can translate into improved clinical outcomes in this high-risk population.

## Data Availability

The original contributions presented in the study are included in the article/Supplementary Material, further inquiries can be directed to the corresponding author/s.
