# Supplementary material for: Effect of stress-induced hyperglycaemia on clinical outcome in paitients with acute ST-segment elevation myocardial infarction undergoing percutaneous coronary intervention
Source: Front Cardiovasc Med. 2026 May 14;13:1763222. doi: 10.3389/fcvm.2026.1763222 (PMC13215925; doi:10.3389/fcvm.2026.1763222)
Supplement: Supplementary file 1 [file Table1.docx]

Supplementary Table S1: Comparison of baseline characteristics between patients with complete and incomplete clinical risk score data

| Variable | Complete data (n = 655) | Incomplete data (n = 163) | P value |
| --- | --- | --- | --- |
| Age (years), mean ± SD | 61.1 ± 13.2 | 60.9 ± 13.4 | 0.862 |
| Male sex, n (%) | 506 (77.3) | 126 (77.3) | 0.998 |
| Hypertension, n (%) | 323 (49.3) | 80 (49.1) | 0.956 |
| Smoking, n (%) | 294 (44.9) | 73 (44.8) | 0.979 |
| Killip class 2-4, n (%) | 102 (15.6) | 25 (15.3) | 0.934 |
| FBG (mmol/L), mean ± SD | 7.74 ± 2.50 | 7.78 ± 2.55 | 0.857 |
| HbA1c (%), mean ± SD | 6.71 ± 1.71 | 6.74 ± 1.74 | 0.841 |
| Gensini score, mean ± SD | 53.6 ± 14.4 | 53.5 ± 14.3 | 0.935 |
| Group allocation, n (%) |  |  | 0.921 |
| - Non-SHG group | 255 (38.9) | 64 (39.3) |  |
| - Diabetes group | 240 (36.6) | 59 (36.2) |  |
| - SHG group | 160 (24.4) | 40 (24.5) |  |

Abbreviations: FBG, fasting blood glucose; HbA1c, glycated hemoglobin; SHG, stress hyperglycemia. Note: No significant differences were observed between the two groups for any variable (all P > 0.05).
